# Long–term outcomes in physical function and quality of life after traumatic thoracolumbar A3/A4 fractures: a comparison of conservative versus surgical management

**DOI:** 10.1007/s00068-026-03135-2

**Published:** 2026-03-12

**Authors:** Anna Silke Sienema, Inge H. F. Reininga, Joost Hoekstra

**Affiliations:** Department of Trauma Surgery, University of Groningen, University Medical Center Groningen, HPC BA13, Hanzeplein 1, Groningen, 9713 GZ The Netherlands

**Keywords:** Thoracolumbar A3/A4 fractures, Patient-reported outcome, Vertebral fractures, Physical functioning, Quality of life

## Abstract

**Purpose:**

To provide insight into the recovery rates of patient-reported outcome measures (PROMs) in terms of physical functioning and health-related quality of life (QoL) in patients with an A3/A4 thoracolumbar vertebral fracture, compared to the general population. Additionally, differences in outcomes between conservatively and surgically treated patients were assessed.

**Methods:**

A retrospective cohort study with cross-sectional follow-up including patients with thoracolumbar A3/A4 vertebral fractures in a level 1 trauma center between 2010 and 2020. SMFA-NL was used to evaluate physical functioning, and EQ-5D was used to assess QoL. Outcomes were compared with normative data from the Dutch population. Patient-reported outcomes and complication rates were reported for each treatment type. Recovery was defined as reaching the lower limit of the 95% confidence interval of the normative data in all outcome measures.

**Results:**

PROMs were available for 98 (37%) of the eligible patients with a median follow-up of 4.5 (IQR = 5.6) years. No significant differences in physical functioning or QoL were found between conservatively and surgically treated patients. The following non-recovery rates were found in the conservatively treated patients: physical functioning = 56–74%, QoL = 48% and in the surgically treated patients: physical functioning = 77–80%, QoL = 45%. Surgically treated patients showed significantly higher complication rates than conservatively treated patients.

**Conclusion:**

Thoracolumbar A3 and A4 fractures are associated with long-term reduced physical functioning and quality of life compared with the general population, regardless of treatment strategy. Both surgically and conservatively treated patients showed significantly low recovery rates in comparison with their peers from the general population.

## Introduction

Vertebral fractures are common and carry major consequences [1]. They cause more disability and higher healthcare costs than other musculoskeletal injuries [2]. In younger patients, they typically result from high‑energy trauma (HET), while in older adults they often occur after low‑energy trauma (LET) [3]. About 90% of spinal fractures occur at the thoracolumbar junction [4]. The incidence of thoracolumbar fractures is increasing—from 21.5 per 100,000 inhabitants in 2010 to 24 per 100,000 in 2017 [5], a rise of about 12%. This trend is linked to the growing prevalence of age‑related osteoporosis [6].

Vertebral fractures can lead to severe complications like neurologic deterioration, chronic back pain, and deterioration in physical functioning. All these complications may, in turn, result in disability and loss of quality of life [1, 7]. Vertebral fractures are usually classified according to the AO Spine Classification System. Based on the AO Spine classification, a specific treatment will be started. This treatment could involve surgical stabilization of the affected vertebra or conservative treatment, including pain management, physical therapy, and, if necessary, an orthosis [8].

For some types of vertebral fractures, the treatment plan is obvious. For example, an A0 fracture, where only the processus spinosus has been affected, is almost always treated conservatively. Of the thoracolumbar fractures, 14–17% are classified as A3 or A4 fractures according to the AO Spine Classification System [9]. However, despite being a prevalent type of fracture, the treatment of A3 and A4 thoracolumbar fractures remains a grey area, as the optimal treatment has not yet been established [10]. A3 and A4 fractures are treated surgically as well as conservatively; however, neither therapy has proven itself superior to the other [9, 10].

The meta-analysis by Chou et al. [11] showed no significant differences in outcomes between surgical and conservative treatment for thoracolumbar A3/A4 vertebral fractures. They examined outcomes at least 6 months following fracture regarding pain, as measured by analgesic use or the VAS pain score and physical functioning using the Oswald Disability Index and Roland Morris Questionnaire on Disability (RMQD) [11]. A systematic review and meta-analysis on measurement properties of the ODI and RMDQ showed that both PROMs lack a sufficient level of reliability and validity [12]. Moreover, no standard population norms for the RMDQ and ODI are available. Overall, evidence regarding long-term QoL following A3/A4 thoracolumbar fractures is also lacking. Hence, the aim of this study was to compare the self-reported outcomes of patients on long-term physical functioning and QoL between surgically and conservatively treated patients with an A3 or A4 thoracolumbar vertebral fracture. Valid and reliable PROMs (SMFA and EQ-5D-5 L) were used. For these PROMs, general population norms are available. Hence, this study also aimed to assess whether these patients showed lower levels of recovery of physical functioning and QoL, compared to normative data of the Dutch population.

## Methods

### Patients

The study design was a retrospective cohort study. All adult patients (≥ 18 years of age) who were treated for an A3 or A4 vertebral fracture at the trauma surgery department of the UMCG between 01-01-2010 and 01-05-2020 and had a minimum follow-up of one year following the injury were included. The data from 01-01-2010 until 31-12-2019 had already been collected for previous research. This database consists of 284 patients. This database was complemented with data on patients treated for an A3/A4 fracture between 01-01-2020 and 31-12-2021, totaling an additional 37 patients.

The local medical ethics review board reviewed the methods and waived the need for further approval (METc 2019/606).

### Data collection

Data on patient characteristics, treatment type, and possible complications were gathered from the hospital’s electronic patient records. Patients’ comorbidities were classified according to the Charlson Comorbidity Index, which assigns points to each comorbidity; a higher score indicates more comorbidities [13]. Complications were classified according to the Clavien-Dindo (CD) grading system, ranging from grade 0 to 5 [14]. A score of 0 indicates no complications, whereas a score of 5 indicates complications resulting in the patient’s death. An experienced trauma surgeon reassessed all radiographic images of the included patients to validate the type of fracture. Neurological status was scored according to the AO guidelines ranging from N0-N4 and Nx. N0 indicates no neurologic injury whereas N4 means complete neurologic injury. Nx means that evaluation was not possible [10]. The thoracolumbar AOSpine Injury Score (TL AOSIS) was calculated for all patients. TL AOSIS integrates fracture morphology, neurological status, and clinical modifiers. Each category is assigned a weighted numerical value, resulting in a cumulative score that reflects overall injury severity. The TL AOSIS is designed to support clinical decision making, particularly in guiding operative versus conservative management of thoracolumbar vertebral fractures [10]. Data was collected and stored using REDCap software. REDCap is a secure, web-based software platform to facilitate data acquisition and storage for research purposes [15].

### Patient-reported outcome measures

Patients without cognitive disorders who were still alive at follow-up received a letter with a link to an online questionnaire to assess their long-term physical functioning and quality of life. The functional status of the patients has been evaluated using the Dutch version of the Short Musculoskeletal Function Assessment (SMFA-NL). The SMFA is a patient-reported questionnaire comprising 46 questions and is used to detect differences in functional status among patients with a broad range of musculoskeletal injuries [16]. The SMFA-NL consists of four subscales: lower extremity, upper extremity, mental and emotional problems and ADL (general activities of daily living). The score ranges from 0 to 100, with higher scores indicating better functional status.

To assess patients’ quality of life (QoL), the Dutch EuroQol 5 L (EQ-5D) questionnaire has been used [17]. The EQ-5D consists of five items and is built on five domains that assess patients’ QoL. These domains are: mobility, self-care, usual activities, pain/discomfort, and anxiety/depression. The EQ-5D yields a utility score ranging from 0 to 1, with 0 indicating the worst imaginable health and 1 the best.

Normative data of the SMFA-NL [18] and the EQ-5D [17] were used to determine whether the patient recovered to the expected level of their age group in the general Dutch population. SMFA-NL normative data were further specified by gender. If the patient’s score reached the lower limit of the 95% confidence interval of the normative data, the patient was considered recovered.

### Statistical analysis

Multiple imputation and data analysis were performed using IBM SPSS software version 30.0.0.0 with a significance level of *p* < 0.05. Descriptive statistics have been used to present clinical outcomes and PROMs. For normally distributed data, the mean and standard deviation (SD) have been used; for non-normally distributed data, the median and interquartile range (IQR) have been used, and numbers are represented as frequencies and percentages (%).

To address uncertainty from missing data, we applied multiple imputation for partially completed SMFA subscales and EQ-5D utility scores. In total, 6 patients (6%) had missing items on the SMFA Lower Extremity subscale, 2 (2%) on Upper Extremity, 2 (2%) on Emotional Status, and 7 (7%) on ADL. One patient (1%) had a missing EQ-5D item. Imputation assumed data were missing at random and included gender, Charlson Comorbidity Index (CCI), and available item scores from the respective SMFA subscale or EQ-5D. Five complete datasets were generated using 10 iterations.

To assess differences in functional status and QOL between the surgical and conservative treatment groups, an independent-samples t-test was performed on the SMFA and EQ-5D outcomes. Next, to assess whether there was a difference in non-recovery rate, the non-recovery rate of the surgical group was compared with that of the conservatively treated group using logistic regression analyses. Gender, number of comorbidities and TL AOSIS score were added as potential confounders.

To evaluate differences in SMFA and EQ-5D scores between patients and the general population, independent-samples t-tests were conducted. Additionally, comparisons were made between conservatively and surgically treated patients relative to normative data from the general population using independent-samples t-tests.

In addition, to assess whether there was a difference in complication rate, a chi-square test has been used based on the Clavien-Dindo index. Grades 1 and 2 were defined as minor complications, grade 3 and higher as severe. In case of a positive chi-square, a subgroup analysis was performed. To determine whether the presence of complications was associated with reaching the lower limit of the 95% CI of the norm data of the PROMs outcomes, a chi-square test was performed.

## Results

### Study population

A total of 284 patients with thoracolumbar A3/A4 fractures were identified. The patient characteristics are shown in Table 1.

**Table 1:** patient characteristics

	Total (*n* = 284)	Conservative treatment (*n* = 166)	Surgical treatment (*n* = 118)
Age, median (IQR)	55 (27)	59 (28)	52.5 (26.5)
Male (%)	160 (56)	85 (51)	75 (64)
Multiple spinal fractures (%)	23 (8)	13 (8)	10 (8)
Trauma mechanism (%)			
*Traffic car*	27 (10)	18 (11)	9 (8)
*Traffic motorcycle*	8 (3)	4 (2)	4 (3)
*Traffic bike*	27 (10)	17 (10)	10 (8)
*Traffic pedestrian*	1 (0.3)	0 (0)	1 (1)
*Fall < 3 m*	140 (49)	87 (52)	53 (45)
*Fall > 3 m*	37 (13)	22 (13)	15 (13)
*Other*	43 (15)	18 (11)	25 (21)
Neurologic injury (%)			
*N0*	247 (87)	156 (94)	91 (77)
*N1*	5 (2)	4 (2)	1 (1)
*N2*	12 (4)	4 (2)	8 (7)
*N3*	13 (5)	0 (0)	13 (11)
*N4*	1 (0.3)	0 (0)	1 (1)
*Nx*	1 (0.3)	1 (1)	1 (1)
Type of fracture (%)			
*A3*	136 (48)	104 (63)	32 (27)
*A4*	148 (52)	62 (37)	86 (73)
TL AOSIS, median (IQR)	5 (2)	3 (2)	5 (0)
*0–3 (%)*	124 (44)	100 (60)	24 (20)
*4 (%)*	3 (1)	2 (1)	1 (1)
*≥ 5 (%)*	157 (55)	64 (39)	93 (79)
Surgical details (%)			
*Open monoaxial fixation*	94 (33)		94 (80)
*Open polyaxial fixation*	14 (5)		14 (12)
*Percutaneous minimal invasive*	6 (2)		6 (5)
Other*	3 (1)		3 (3)
Comorbidities (%)			
*CCI 0–1*	136 (48)	73 (44)	63 (53)
*CCI 2–5*	123 (43)	75 (45)	48 (41)
*CCI > 5*	25 (9)	18 (11)	7 (6)

Charlson Comorbidity Index score (CCI)

*Other surgical techniques include: vertebral body stent, spondylodesis and corpectomy

### Availability of patient-reported outcome measures

**Fig. 1:**
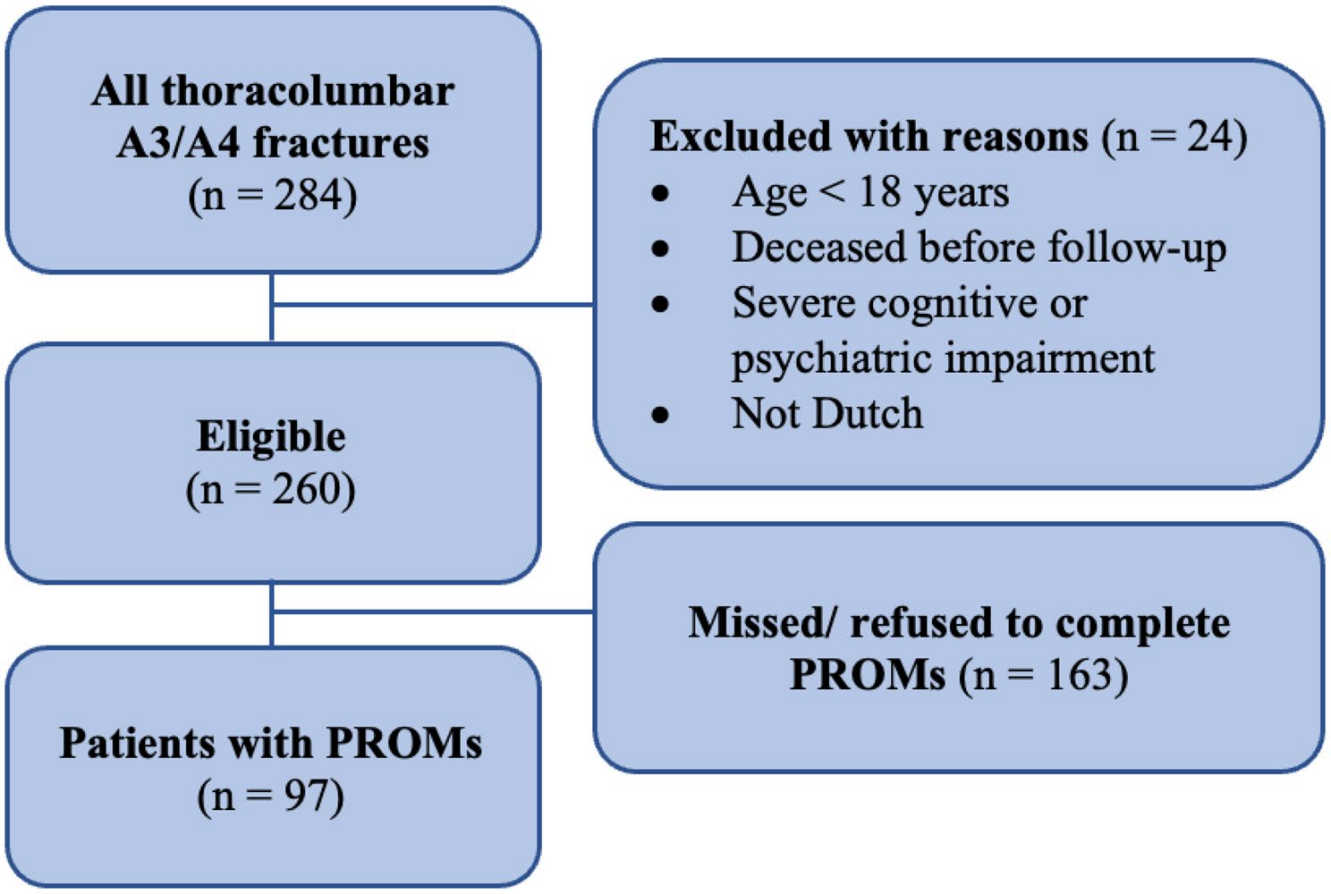
Flowchart depicting the availability of PROMs

A flowchart illustrating patient inclusion and the availability of PROMs is shown in Fig. 1. Of the 284 patients with a thoracolumbar A3/A4 vertebral fracture, 260 (92%) were deemed eligible to participate by filling out PROMs. Among those eligible, 97 (37%) completed PROMs with a median follow-up duration of 4.5 years (IQR = 5.6).

### Non-response analysis

We performed a non-response analysis. This analysis showed no significant differences regarding age at time of fracture, age at time of follow-up, gender and neurological status. Follow-up duration (i.e. time between fracture and invitation to fill in PROMs) was however significantly longer in the non-responder group, compared to the responder group: 5 (IQR 5) vs. 4 (IQR 4) years, *p* = 0.03.

### Complication rate

The analysis of complication rates showed that surgically treated patients had significantly higher complication rates than conservatively treated patients (Table 2).

**Table 2:** Complication rates of conservatively versus surgically treated patients

	Total	Conservative treatment	Surgical treatment	*P*-value
No complications, *n* (%)	244 (87)	154 (93)	90 (80)	0.002
Minor complications, n (%)	27 (10)	11 (7)	16 (14)	
Major complications, n (%)	8 (3)	1 (1)	7 (6)	

Table 3 shows the pooled means of the SMFA subscales and EQ-5D scores for all patients, compared with those of the norm population. Notably, patients treated for a thoracolumbar A3/A4 fracture scored significantly lower on all subscales, except for the SMFA upper extremity subscale.

**Table 3:** Mean SMFA subscale scores and EQ-5D utility score compared to normative data of the Dutch population

	Patient	Norm	*P*-value
SMFA score			
Lower extremity	83.4	87.7	**0.030**
Upper extremity	95.3	94.3	0.417
Mental and Emotional problems	74.0	79.0	**0.004**
ADL	75.6	85.8	**< 0.001**
EQ-5D	0.798	0.860	**0.004**

Imputed data are presented

To assess differences in means and significance between surgically treated and conservatively treated patients, a subgroup analysis was conducted. Table 4 shows the pooled means of the SMFA and EQ-5D scores per treatment type, compared with the normative data for the Dutch population.

**Table 4:** Mean SMFA subscale scores and EQ-5D utility score per treatment, compared to normative data of the Dutch population

	Conservative treatment			Surgical treatment		
	Patient	Norm	*P*-value	Patient	Norm	*P*-value
SMFA-score						
Lower extremity	83.9	87.4	0.120	82.7	88.2	0.135
Upper extremity	95.8	94.0	0.205	94.5	94.8	0.888
Mental and Emotional problems	74.8	78.6	0.085	72.7	79.7	**0.018**
ADL	76.3	85.3	**< 0.001**	74.7	86.5	**0.004**
EQ-5D	0.815	0.86	0.060	0.77	0.86	**0.027**

Imputed data are presented

These results show that patients in both treatment groups scored significantly lower on the SMFA ADL subscale and EQ-5D than the norm population. The score on the mental and emotional problems subscale of the SMFA, however, was significantly lower in the surgically treated group but not in the conservatively treated group.

**Fig. 2:**
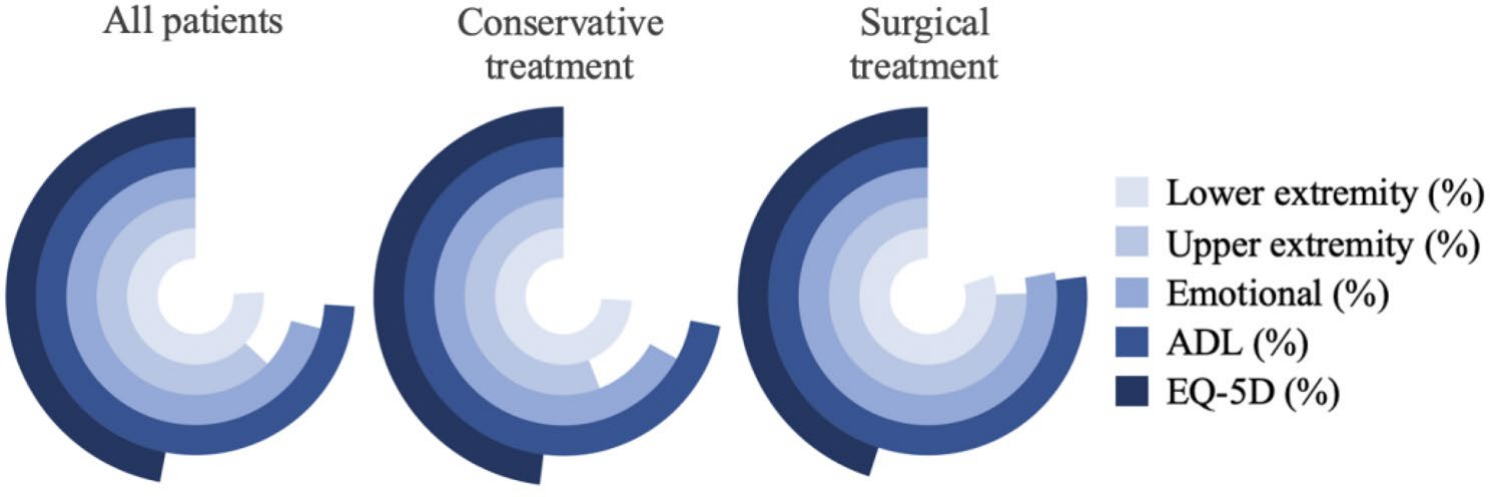
Percentage of patients deemed not recovered per treatment group

The number of patients not recovered in each subscale, i.e., not reaching the lower limit of the 95% CI for the respective norm data, is shown in Table 5; Fig. 2, along with the comparison of these non-recovery rates between the conservative and surgical treatment groups.

**Table 5:** Number (%) of patients that did not recover on the SMFA subscales and EQ-5D and logistic regression analyses of the differences in non-recovery rates

	Total	Conservative treatment	Surgical treatment	*P*-value	OR (95% CI)**
SMFA-score					
Lower extremity, n (%)	71 (76)	43 (74)	28 (80)	0.919	0.94 (0.29–3.03)
Upper extremity, n (%)	61 (63)	33 (56)	28 (80)	0.368	0.64 (0.24–1.71)
Mental and Emotional problems, n (%)	69 (71)	40 (67)	29 (78)	0.562	0.56 (0.20–1.62)
ADL, n (%)	68 (74)	41 (72)	27 (77)	0.902	0.90 (0.30–2.73)
EQ-5D, n (%)	46 (47)	29 (48)	17 (45)	0.926	1.04 (0.42–2.60)

Imputed data are presented

* P-value of comparison of non-recovery rates between surgical and conservative treated patients

** Logistic regression analysis corrected for TL AOSIS score

It was observed that many patients did not recover from a thoracolumbar A3/A4 fracture. Comparing recovery rates between surgically and conservatively treated patients did not show significant differences between the two treatment groups.

## Discussion

This study presents patient-reported outcomes on physical functioning and health-related quality of life (QoL) in individuals treated for thoracolumbar A3/A4 fractures, either conservatively or surgically, at a median follow-up of 4.5 (IQR = 5.6) years. No statistically significant differences were observed between the conservative and surgical treatment groups in levels of physical functioning or health-related quality of life. These findings suggest that, despite differences in treatment approach, long-term patient-reported outcomes are comparable across both groups.

Compared with normative data from the Dutch population, patients in both treatment groups reported significantly lower levels of physical functioning and QoL. Specifically, impairments were noted in daily activities, lower extremity function, mental and emotional well-being, and overall QoL. On the EQ-5D, 47% of patients failed to reach the normative threshold. In comparison, non-recovery rates on the SMFA subscales were even higher: 76% for lower extremity, 63% for upper extremity, 71% for mental and emotional problems and 74% for ADL. No significant differences in non-recovery rates were observed between the two treatment groups, although these results should be interpreted with caution because of the small sample sizes.

A significant difference in complication rates was observed between the treatment groups: patients treated conservatively experienced notably fewer minor and major complications than those who underwent surgical treatment. This finding aligns with expectations, as surgical treatment is inherently more invasive, which may increase the risk of complications. However, it is essential to note that conservative treatment is not suitable for all patients, particularly those with neurological deficits or unstable fracture patterns, where surgical management remains necessary.

### Patient-reported physical functioning

Several studies have compared surgical and conservative treatment approaches for A3/A4 thoracolumbar fractures using patient-reported outcome measures (PROMs), consistently showing that long-term outcomes are broadly comparable between the two strategies. The systematic review by Chou et al. [11] reported no significant differences in physical functioning between surgical and conservative treatments after 6 months of follow-up, based on scores from the patient-reported Roland-Morris Disability Questionnaire (RMDQ) and Oswestry Disability Index (ODI) [11]. Although these results are in line with our findings, they included all thoracolumbar burst fractures and not only the A3/A4 fractures. The extensive international AO Spine cohort study by Dvorak et al. [19] further confirmed that both treatment modalities for thoracolumbar A3/A4 fractures result in comparable long-term disability, as assessed using standardized instruments such as the Oswestry Disability Index (ODI). However, their sample size was relatively small. Moreover, both the RMDQ and ODI lack adequate reliability and validity [12].

In contrast, our study utilized the SMFA, offering a broader and more detailed assessment of physical functioning. The SMFA demonstrates sufficient measurement properties. It captures a wider range of musculoskeletal limitations and daily activity impairments than the ODI or RMDQ, providing a more comprehensive view of long-term recovery in this patient population. Collectively, these studies underscore that thoracolumbar A3/A4 fractures have a lasting impact on patients’ physical and emotional functioning, reinforcing the notion that full functional recovery to pre-injury levels is uncommon, irrespective of treatment modality.

### Health-related quality of life

To date, limited research has explored the impact of thoracolumbar A3/A4 fractures on quality of life, particularly when comparing outcomes between surgical and conservative treatment approaches. Vialle et al. [20] reported similar outcomes comparing conservative treatment to surgical treatment in patients with thoracolumbar A3/A4 fractures as in our study. However, this study reports a low sample size of 16 patients with a 2.5-year follow-up. They also presented raw EQ-5D scores rather than utility index conversions in their primary analysis. Failing to convert EQ-5D scores to utility indices results in less standardized comparisons across patient groups or interventions. In comparison, our study included a relatively larger sample size and utilized EQ-5D utility scores, thereby enhancing the standardization and clinical relevance of the findings.

### Comparison with normative data of the general Dutch population

The present study is, to our knowledge, the first to assess recovery following A3/A4 thoracolumbar fractures using normative data from a general population as a reference point. This approach provides a more objective benchmark for evaluating outcomes and highlights the extent to which patients fall short of population norms, regardless of treatment strategy. Our findings further demonstrate that most patients fail to reach even the lower limit of the 95% confidence interval of the normative data of their peers. Moreover, previous work by De Graaf et al. [21] showed that pre-injury PROM scores in trauma patients are generally higher than normative population values, suggesting that our findings likely represent a conservative estimate of incomplete recovery. This highlights the need for continued research to optimize treatment strategies and better understand why patients fail to reach this point, enabling the development of more effective treatments.

### Complication rate

This study showed significantly higher complication rates after surgical treatment of thoracolumbar A3/A4 fractures, compared to conservative treatment. These findings correspond with recent evidence reporting a higher incidence of procedure-related morbidity following operative stabilization of thoracolumbar burst fractures. A retrospective multicenter analysis by Wang et al. [22] demonstrated that postoperative complications occurred in 10–14% of surgically treated patients, most commonly wound infections, hardware loosening, and pulmonary complications, while conservatively managed patients experienced fewer adverse events overall. Similarly, a population-based registry study by Aono et al. [23] found that although surgery provided superior radiological correction, it was associated with a twofold higher risk of perioperative complications and prolonged hospital stay. More recently, Chen et al. [24] reported comparable findings in a prospective cohort, noting that 12% of surgical patients developed procedure-related complications compared to only 5% in the nonoperative group.

Overall, these findings confirm that while modern surgical techniques have improved safety, complication rates remain higher compared to conservative treatment. This reinforces the need for careful consideration when opting for surgery, balancing potential risks with the comparable long-term outcomes of conservative management. In conclusion, patients who sustained a thoracolumbar A3/A4 fracture and were treated conservatively or surgically have similar outcomes in terms of physical functioning and quality of life. Most patients do not recover to the level of their peers from the general population and the choice for surgical treatment should be made carefully because of the higher incidence of complications.

## Strengths and limitations

A key strength of this study is the use of valid and reliable patient-reported outcome measures, including both the SMFA and the EQ-5D, to capture not only the functional consequences of thoracolumbar A3/A4 fractures in daily living but also health-related quality of life. To our knowledge, this is the first study to compare quality of life between conservatively and surgically treated patients with thoracolumbar A3/A4 fractures using the EQ-5D utility scores. Furthermore, it is the first to evaluate patient-reported outcome measures (PROMs) in this population against normative data from the general Dutch population, providing a robust reference point for interpreting recovery and residual disability.

Nevertheless, several limitations must be acknowledged. The cross-sectional study design may introduce selection bias, as treatment decisions are often influenced by neurological status and fracture characteristics. The sample size, while sufficient to reveal meaningful differences in physical functioning and QoL, may be underpowered to detect more subtle differences between treatment groups. The surgically treated group was analyzed as a single entity despite heterogeneity in surgical techniques. As a result, treatment specific effects and complication profiles could not be evaluated, and the results should be interpreted with caution. Furthermore, the cross-sectional nature of the analysis prevents a complete understanding of recovery trajectories over time. Another weakness of this study design is that data collection depended on the completeness of data recorded in the electronic patient files. As a result, relevant data may have been missed. Furthermore, a response rate of 37% might seem low but is not uncommon in trauma populations, partly because a proportion of patients have died during long-term follow-up. This rate is comparable to previous studies on outcome following vertebral fractures, such as the study by Filiberto et al. [7], who reported a response rate of 35% after a mean follow up of 5.7 years, and the study by Vialle et al. [20], in which only 16 of 59 patients (27%) completed the 2.5 year follow up questionnaire. The non-response analysis showed no clinically significant differences between the responders and non-responders in terms of age, gender and neurological status. A statistically significant difference was found in the median follow-up duration between the responders and non-responders (4 vs. 5 years). However, we consider this statistically significant difference of one year not clinically relevant, as in general, patient-reported physical functioning and quality of life tends to be stable after at least one year after the fracture, and we consider it highly unlikely that the patient-reported physical functioning and quality of life suddenly declines after 4 years of follow-up. Finally, unmeasured confounding factors such as pre-injury health status socioeconomic determinants, and adherence to rehabilitation may have influenced patient-reported outcomes.

## Implications for clinical practice

The findings highlight that despite advances in both conservative and surgical management, many patients with thoracolumbar A3/A4 fractures do not achieve the physical functioning and quality-of-life levels of their peers from the general population. Importantly, no significant differences were observed between surgical and conservative treatments, suggesting that surgical intervention does not guarantee superior long-term recovery.

However, surgical treatment remains necessary in certain cases, particularly with neurological involvement or unstable fracture patterns. These results underscore the importance of patient education and shared decision-making in treatment planning, with realistic communication about expected outcomes and the likelihood of residual limitations. Clinicians should also consider early integration of comprehensive rehabilitation and psychosocial support, as functional impairment and emotional challenges persist in a substantial proportion of patients.

## Directions for future research

Future studies should adopt prospective, multicenter designs with larger cohorts to better delineate which patient subgroups may benefit most from surgical versus conservative management. Longitudinal studies are needed to track recovery trajectories and identify predictors of incomplete recovery, both functional and psychosocial. Further research should also focus on understanding *why* many patients fail to reach the level of their peers, whether due to persistent pain, biomechanical limitations, psychosocial burden, or insufficient rehabilitation strategies.

## Conclusion

This study showed that thoracolumbar A3/A4 fractures are associated with reduced long-term physical functioning and quality of life compared with the general population. While complication rates were higher in surgically managed patients, no significant differences in physical functioning and health-related quality of life were observed between treatment strategies, indicating that surgery does not ensure superior recovery. Importantly, a substantial proportion of patients fail to reach the levels of their peers, underlining the need for further research to better understand the rehabilitation process, fracture morphology, and the specific factors contributing to persistent problems. Future prospective studies are essential to identify these determinants and develop targeted interventions to improve long-term outcomes.

## Data Availability

Data sets generated during the current study are available from the corresponding author on reasonable request.
